# On the informative value of community‐based indoor radon values in relation to lung cancer

**DOI:** 10.1002/cam4.70126

**Published:** 2024-08-28

**Authors:** Albert Rosenberger, Heike Bickeböller, David C. Christiani, Geoffrey Liu, Matthew B. Schabath, Luisa F. Duarte, Loic Le Marchand, Christopher Haiman, Teresa Landi, Dario Consonni, John K. Field, Michael P. A. Davies, Demetrios Albanes, Adonina Tardón, Guillermo Fernández‐Tardón, Gad Rennert, Christopher I. Amos, Rayjean J. Hung

**Affiliations:** ^1^ Department of Genetic Epidemiology University Medical Center, Georg‐August‐University Göttingen Göttingen Germany; ^2^ Department of Environmental Health Harvard T.H. Chan School of Public Health and Massachusetts General Hospital/Harvard Medical School Boston Massachusetts USA; ^3^ Medical Oncology and Medical Biophysics Princess Margaret Cancer Centre Toronto Ontario Canada; ^4^ Medicine and Epidemiology, Dalla Lana School of Public Health University of Toronto Toronto Ontario Canada; ^5^ Department of Cancer Epidemiology H. Lee Moffitt Cancer Center and Research Institute Tampa Florida USA; ^6^ Epidemiology Program University of Hawaii Cancer Center Honolulu Hawaii USA; ^7^ Center for Genetic Epidemiology, Department of Population and Public Health Sciences, Keck School of Medicine University of Southern California Los Angeles California USA; ^8^ Division of Cancer Epidemiology and Genetics National Cancer Institute, US National Institutes of Health Bethesda Maryland USA; ^9^ Epidemiology Unit Fondazione IRCCS Ca' Granda Ospedale Maggiore Policlinico Milan Milan Italy; ^10^ Department of Molecular and Clinical Cancer Medicine Roy Castle Lung Cancer Research Programme, The University of Liverpool Liverpool UK; ^11^ Faculty of Medicine University of Oviedo, ISPA and CIBERESP Oviedo Spain; ^12^ Clalit National Cancer Control Center and Department of Community Medicine and Epidemiology at Carmel Medical Center and Technion Faculty of Medicine Haifa Israel; ^13^ Dan L Duncan Comprehensive Cancer Center Baylor College of Medicine Houston Texas USA; ^14^ Lunenfeld‐Tanenbaum Research Institute, Sinai Health System University of Toronto Toronto Ontario Canada; ^15^ Dalla Lana School of Public Health University of Toronto Toronto Ontario Canada

**Keywords:** histological subtypes, lung cancer, radiation, smoking

## Abstract

**Background:**

Radon is a radioactive gas and a major risk factor for lung cancer (LC).

**Methods:**

We investigated the dose–response relationship between radon and LC risk in the International Lung Cancer Consortium with 8927 cases and 5562 controls from Europe, North America, and Israel, conducted between 1992 and 2016. Spatial indoor radon exposure in the residential area (sIR) obtained from national surveys was linked to the participants' residential geolocation. Parametric linear and spline functions were fitted within a logistic regression framework.

**Results:**

We observed a non‐linear spatial‐dose response relationship for sIR < 200 Bq/m^3^. The lowest risk was observed for areas of mean exposure of 58 Bq/m^3^ (95% CI: 56.1–59.2 Bq/m^3^). The relative risk of lung cancer increased to the same degree in areas averaging 25 Bq/m^3^ (OR = 1.31, 95% CI: 1.01–1.59) as in areas with a mean of 100 Bq/m^3^ (OR = 1.34, 95% CI: 1.20–1.45). The strongest association was observed for small cell lung cancer and the weakest for squamous cell carcinoma. A stronger association was also observed in men, but only at higher exposure levels. The non‐linear association is primarily observed among the younger population (age < 69 years), but not in the older population, which can potentially represent different biological radiation responses.

**Conclusions:**

The sIR is useful as proxy of individual radon exposure in epidemiological studies on lung cancer. The usual assumption of a linear, no‐threshold dose–response relationship, as can be made for individual radon exposures, may not be optimal for sIR values of less than 200 Bq/m^3^.

## Graphical abstract

Spatial indoor radon levels are useful in epidemiological studies of lung cancer as a proxy for individual indoor radon exposure. Indoor radiation, expressed as a community average, is associated with lung cancer through a J‐shaped profile, with the lowest risk occurring in areas with average indoor radon levels of about 58 Bq/m^3^.

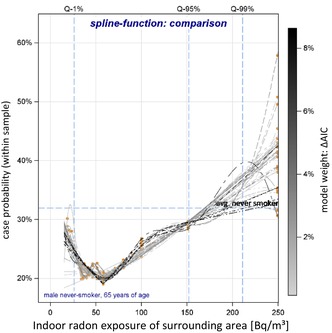

## 1 INTRODUCTION

Lung cancer (LC) is the leading cause of cancer‐related deaths worldwide^1^, ^2^ with five‐year survival remaining low at 13%–18%.^3^ Higher radioactivity concentrations of the naturally occurring, radioactive noble gas radon (^222^Rn and ^220^Rn), given in Becquerel per cubic meter (Bq/m^3^), can accumulate in cavities, such as mines, homes, or in the lungs, with radiation exposure coming mainly from decay products such as polonium, lead, and bismuth.^4^, ^5^, ^6^ Radon and its progenies are henceforth referred to as radon. An increased risk of LC associated with the inhalation of radon, has been consistently demonstrated in several studies of indoor exposure in dwellings (low‐dose environmental exposure) as well as for uranium miners (high‐dose occupational exposure).^4^, ^5^, ^7^, ^8^, ^9^, ^10^, ^11^, ^12^ Radon causes 3 to 12% of all lung cancer cases,^13^ is considered a major risk factor for lung cancer, and was hence declared a human carcinogen by the US Environmental Protection Agency (USEPA) and International Agency for Research on Cancer (IARC).^4^, ^14^, ^15^, ^16^ Upper tolerance limits for buildings (so called radon action levels), ranging from 100 Bq/m^3^ (e.g., WHO) to 300 Bq/m^3^ (European Directive 2013/59/Euratom) were set by governmental authorities to counteract high exposures.^17^

According to large meta‐analyses and systematic reviews, the excess relative risk/odds ratio (ERR/EOR) increases linearly with indoor radon concentration (IRC), which is known as the linear‐no‐threshold (LNT) hypothesis.^9^, ^10^, ^11^, ^12^, ^18^ Average EOR estimates ranged between 8.4% (Darby et al.^9^) and 14% (Malinovsky et al.^18^) per 100 Bq/m^3^. (see Data S1). However, radon‐related lung cancer has been reported to be most prevalent in small cell lung cancer (SCLC) or adenocarcinoma (AdenoLC), while no significant association was found in squamous cell carcinoma (SqCLC).^9^, ^19^, ^20^ Also, an interaction between cigarette smoking and low‐dose radon exposure is generally considered to be more than additive, albeit sub‐multiplicative.^12^, ^21^ This makes radon more dangerous among smokers, but it is also considered one of the most important risk factors for lung cancer among non‐smokers.^4^, ^21^

Estimates of residential/indoor radon exposure (averaged radioactivity concentrations) on the scale of counties or municipalities exist in many European countries, Israel, Canada, the United States, and other countries. The world average IRC has been estimated to be approximately 39 Bq/m^3^, with large variations between geographic regions and countries (for example, averages range from 11 Bq/m^3^ in Australia and to 140 Bq/m^3^ in the Czech Republic and Mexico).^4^, ^22^, ^23^ Such estimates were used to create online resources, such as the European Indoor Radon Map and the EPA Map of Radon Zones.^24^, ^25^

To investigate the estimation of the risk for LC from very low‐dose residential radon exposure based on publicly available exposure levels, we performed an analysis using data from the International Lung Cancer Consortium (ILCCO). Possible modification of the effect by smoking, gender, age and histological subtype as reported by others was investigated. The main objective of this analysis was to assess the association between spatial indoor radon exposure (sIR) and LC risk based on the residential addresses of cases and controls in ILCCO studies.

## 2 METHODS

We obtained regional indoor radon exposure (averaged radioactivity concentrations, IRC) data from representative, cross‐regional, or nationwide surveys in dwellings in the USA, Canada, Israel, and the United Kingdom (Table S2). sIR data for Spain were extracted from the European Indoor Radon Map.^24^ The residential location at the time of diagnosis of each participant of one of five ILCCO studies (CAPUA, EAGLE, HSPH, NICCC‐LCA, MSH‐PMH, see Tables S1 and S3 in the supporting information) was blindly linked to the mean spatial indoor radon exposure in the surrounding area (sIR). In total, 8927 patients with incident, pathologically confirmed, first primary lung cancer (cases) and 5562 control subjects were included (For details, see Data S1, Section 2.1–2.4). The participating studies of ILCCO are individually described in the supplement of McKay et al.^26^

### 2.1 Statistical analysis

#### 2.1.1 Proof of concept/comparison of odds ratios

We estimated ORs and EORs for exposure categories that were as comparable as possible to those reported in previous studies.^9^, ^10^, ^11^ Most of these bins were 25, 50, or 100 Bq/m^3^ wide. The bottom bin served as a reference (<25 Bq/m^3^ resp. <150 Bq/m^3^).

Logistic regression models were fitted to estimate the association, adjusted for sex, age, smoking (type: never, former, and current smoker; age at smoking initiation; time since smoking cessation; pack‐years), and conditional on the study site. We reported ORs relative to an exposure level of less than 25 Bq/m^3^ (as in Darby et al. and Krewski et al.) and relative to the level of 50–75 Bq/m^3^ (as this appeared to cover the exposure with the lowest LC risk).

#### 2.1.2 Parametric risk models

We further fit the linear non‐threshold (LNT) model, as it is widely considered preferable. We added the fit of a logistic regression model (logit), a linear shifted non‐threshold model (LNT+), a linear threshold models (LT), and a linear mirror‐point model (LMP = LT+) to allow for an exposure threshold (see Data S1, section 2.5). The ORs/EORs derived from these models were plotted and compared with observed ORs.

#### 2.1.3 Spline model

To assess possible non‐linearity, we applied spline functions for the sIR to the data. Because the choice of the pre‐defined internal knots and the assumptions of the spline models can change the model fit, we averaged over several fitted splines with different settings.^27^ The goodness of model (spline) fit was finally assess by the Akaike information criterion (AIC). The difference in AIC of models with and without spline for sIR (ΔAIC) was used to weight the splines in averaging.

The lowest risk exposure level (LRE) was determined as the average of the local minimum points of the splines (with a 95% prediction intervals PI). Odds ratios (${\text{OR}}_{m,\mathrm{sIR}}$) were estimated using LRE as a reference (see Data S1, Section 2.6). SAS software (version 9.4) was used for the data analysis.

## 3 RESULTS

### 3.1 Study population characteristics

In total, 8927 LC cases and 5562 controls (*n* = 14,489) were included in this analysis (Table 1). As expected, there is a higher percentage of smokers in cases compared to controls. The majority of the patients were male. The most common histological subtype of LC was adenocarcinoma (55%) followed by SqCLC (25%). sIR‐values between 4 and 835 Bq/m^3^ were assigned, with a median of 71.1 Bq/m^3^, with only 2% (*n* = 227) of subjects with a sIR >200 Bq/m^3^ assigned. The median sIR in cases was about 10 Bq/m^3^ higher than in controls (74.0 vs 64.8 Bq/m^3^).

**TABLE 1:** Sample characteristics of cases and controls of participating ILCCO studies.

	All	Controls		LC cases	
	*n*	*n*	%	*n*	%
Total	14,489	5562	100% (38%)	8927	100% (62%)
Source study					
CAPUA	1683	827	15% (49%)	856	10% (51%)
EAGLE	3856	1983	36% (51%)	1873	21% (49%)
HSPH	5858	1720	31% (29%)	4138	46% (71%)
NICCC‐LCA	1184	525	9% (44%)	659	7% (56%)
MSH‐PMH	1908	507	9% (27%)	1401	16% (73%)
Smoking status					
Never smokers	2777	1888	34% (68%)	889	10% (32%)
Former smokers	6479	2495	45% (39%)	3984	45% (61%)
Current smokers	5233	1179	21% (23%)	4054	45% (77%)
Sex					
Male	9023	3601	65% (40%)	5422	61% (60%)
Female	5466	1961	35% (36%)	3505	39% (64%)
Histological subtype					
AdenoLC	–	–	–	4890	55%
SqCLC	–	–	–	2210	25%
SCLC	–	–	–	730	8%
LCLC	–	–	–	408	5%
Unknown	–	–	–	689	8%

		Mean	Median	Range	Mean	Median	Range
Pack years [py]		20.6	10.0	0–260	46.9	42.9	0–363
Never smokers		–	–	–	–	–	–
Former smokers		27.2	20.8	0.01–211.5	46.4	40.0	0.02–272
Current smokers		39.4	35.0	0.05–260.0	57.7	51.0	0.03–363
sIR [Bq/m^3^]		84.3	64.8	7–853	92.9	74.0	4–835
CAPUA		96.4	89.3	13.0–287.2	92.9	89.3	13.0–137.8
EAGLE		63.3	64.2	21.2–249.2	64.6	64.3	23.8–249.2
HSPH		127.0	152.0	11.0–596.0	121.6	144.0	4.0–835.0
NICCC‐LCA		45.0	42.9	31.8–72.2	45.5	42.9	33.4–79.0
MSH‐PMH		52.1	50.3	39.5–154.1	67.6	55.5	27.5–258.7
Age (years)		63.4	65.0	19–96	65.7	66.8	22–95

*Note*: % column‐% (row‐%).

### 3.2 Comparison of odds ratios

To compare the estimates of the ILCCO data with previously published categorized estimates, we grouped the sIR into exposure bins, with the lowest bin as the reference (<25 Bq/m^3^). We observed an increased risk of overall LC in the sIR categories >75 Bq/m^3^. The observed bin‐wise ORs were generally comparable to those reported by Krewski et al.,^10^ Darby et al.,^9^ and Lubin et al.^11^ (Table 2). This suggests that the information content of the sIR values is comparable to that of the IRC values used previously.

**TABLE 2:** Comparison of OR with previously published estimates.

sIR category^b^	Cses	Ccntrol	ILCCO studies				Darby et al.		Krewski et al.		Lubin et al.	
			$\bar{\mathrm{sIR}}$	ORR^a^	95% CI	*p*‐value^d^	OR	95% CI	OR	95% CI	OR	95% CI
Lowest exposure class as reference												
<25 Bq/m^3^ (Ref.)	83	48	18 Bq/m^3^	1.00	reference		1.00	reference	1.00	reference	⌈	⌈
25‐< 50 Bq/m^3^	1302	1055	42 Bq/m^3^	0.98	0.64–1.51	0.9567	1.06^c^	0.98–1.15	1.13^c^	0.94–1.31	|	|
50‐< 75 Bq/m^3^	3171	2195	62 Bq/m^3^	1.00	0.66–1.51	0.9865	1.03^c^	0.96–1.10	1.05^c^	0.86–1.27	1.00	reference
75‐< 100 Bq/m^3^	1142	811	88 Bq/m^3^	1.37	0.89–2.09	0.1436	⌊	⌊	1.14^c^	0.90–1.45	|	|
100‐ < 150 Bq/m^3^	1912	886	131 Bq/m^3^	1.26	0.84–1.91	0.2563	1.20^c^	1.08–1.32	1.22^c^	0.95–1.56	⌊	⌊
150‐ < 200 Bq/m^3^	1166	491	156 Bq/m^3^	1.21	0.80–1.84	0.3580	⌊	⌊	1.19^c^	0.86–1.66	1.42^c^	1.00–2.00
200‐ < 250 Bq/m^3^	75	30	213 Bq/m^3^	1.82	0.98–3.38	0.0577	⌈	⌈	⌈	⌈	1.13^c^	0.80–1.27
250‐ < 300 Bq/m^3^	43	31	269 Bq/m^3^	0.78	0.40–1.53	0.4838	1.18^c^	0.99–1.42	|	|	1.27^c^	0.80–1.90
300‐ <400 Bq/m^3^	29	14	⌈	0.87	0.40–1.90	0.7371	⌊	⌊	1.29^c^	0.93–1.80	⌈	⌈
400‐ < 800 Bq/m^3^	4	1	337 Bq/m^3^	|	|	|	1.43	1.06–1.92	|	|	1.52	1.10–2.20
>800 Bq/m^3^	–	–	⌊	⌊	⌊	⌊	2.02	1.24–3.31	⌊	⌊	⌊	⌊
Selected exposure class as reference												
<25 Bq/m^3^	83	48	18 Bq/m^3^	0.99	0.65–1.50	0.9865	0.97^c^	0.91–1.04	0.95^c^	0.79–1.16	⌈	⌈
25‐< 50 Bq/m^3^	1302	1055	42 Bq/m^3^	0.98	0.85–1.12	0.8226	1.03^c^	0.95–1.12	1.08^c^	0.90–1.25	|	|
50‐< 75 Bq/m^3^ (Ref.)	3171	2195	62 Bq/m^3^	1.00	reference	–	1.00	reference	1.00	reference	1.00	reference
75‐< 100 Bq/m^3^	1142	811	88 Bq/m^3^	1.36	1.18–1.56	<0.0001	⌊	⌊	1.09^c^	0.86–1.38	007C	|
100‐< 150 Bq/m^3^	1912	886	131 Bq/m^3^	1.26	1.11–1.43	0.0004	1.17	1.05–1.28	1.16^c^	0.90–1.49	⌈	⌈
150‐< 200 Bq/m^3^	1166	491	156 Bq/m^3^	1.21	1.03–1.41	0.0164	⌊	⌊	1.13^c^	0.82–1.58	1.42	1.00–2.00
200‐< 250 Bq/m^3^	75	30	213 Bq/m^3^	1.81	1.12–2.94	0.0154	⌈	⌈	⌈	⌈	1.13^c^	0.80–1.27
250‐< 300 Bq/m^3^	43	31	269 Bq/m^3^	0.78	0.45–1.34	0.3807	1.15^c^	0.96–1.38	|	|	1.27^c^	0.80–1.90
300‐<400 Bq/m^3^	29	14	⌈	0.87	0.44–1.71	0.6913	⌊	⌊	1.23^c^	0.89–1.71	⌈	⌈
400‐< 800 Bq/m^3^	4	1	337 Bq/m^3^	|	|	|	1.39	1.03–1.86	|	|	1.52^c^	1.10–2.20
>800 Bq/m^3^	–	–	⌊	⌊	⌊	⌊	1.96	1.20–3.21	⌊	⌊	⌊	⌊
Total	8927	5562										

*Note*: Becquerel (Bq) is the radioactivity unit. This value corresponds to the average number of atomic nuclei that decay radioactively per second. Radioactivity concentration is the amount of radioactivity per unit volume and is given here in Bq/m^3^.

^a^ Adjusted for study site, sex, age, and smoking.

^b^ Categories were chosen to be as comparable as possible to published meta‐analyses.

^c^ Previously published OR contained in 95% confidence interval based on ILCCO study data.

^d^ Two‐sided.

### 3.3 Parametric risk models

Fitting parametric models (Data S1, Section 2.5), we estimated adjusted EORs, based on the linearity assumption, between 0.51 (LNT: 95% CI: 0.37–0.80) and 0.62 (LT: 95% CI: 0.44–0.87) per 100 Bq/m^3^ (see Data S1, Sections 3). These estimates are approximately five times larger than comparable previous estimates, which may be due to the different quantification of IRC and sIR exposure.

**FIGURE 1:**
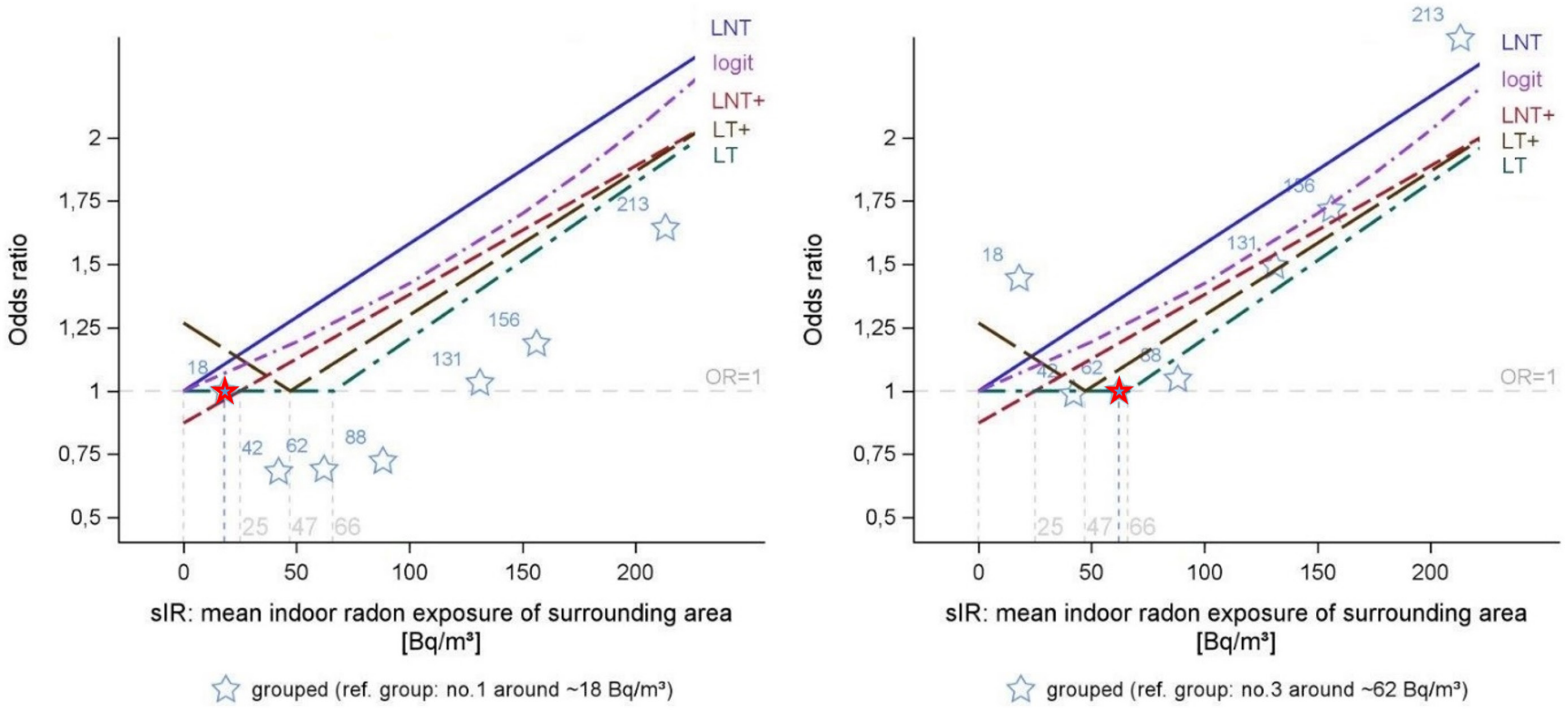
Fit of parametric models. Stars indicate the point estimates of ORs adjusted for study site, sex, age, and smoking (type: Never, former, and current smoker; age at smoking initiation; time since smoking cessation; pack years) by exposure group, the mean sIR is given (see Table 2); the reference exposure class is printed in red (therefore OR = 1). Left panel: Adjusted ORs for exposure groups with reference category “4–<25 Bq/m^3^”; right panel: Adjusted ORs for exposure groups with reference category “50–<75 Bq/m^3^”; LNT linear non‐threshold model; LNT+ linear shifted non‐threshold model; LT linear threshold model; LT+ linear mirror point model; logit logistic regression model.

Figure 1 shows the fit of the parametric models with ORs relative to the lowest exposure bin of 4 to <25 Bq/m^3^, which demonstrated a deviation from the LNT model (see Figure 1, left panel). When fitting LNT+, LT, and LT+, hence allowing for a lower exposure threshold, we observed a shift in the LRE to 25, 47, or 66 Bq/m^3^. ORs for sIR categories relative to the bin of 50 to <75 Bq/m^3^ were visually consistent with these three models (see Figure 1, right panel).

### 3.4 Spline model

To overcome the limitation of the linearity assumption of the parametric models, we fitted a logistic regression model with spline functions for sIR. The spline functions were averaged by weighting their goodness of fit (ΔAIC).

### 3.5 Overall lung cancer

**FIGURE 2:**
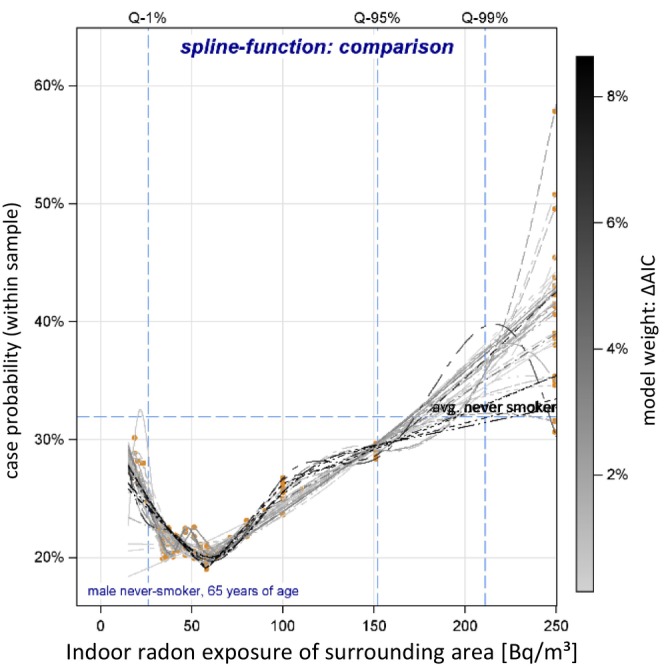
Estimated spline function for the case‐probability (ΔAIC‐ weighted): Overall LC. Spline functions (gray to black lines) are highlighted according to the goodness of model fit measured as ΔAIC, relative to the based model (used as model weights for model averaging); radon exposure: Q‐1%: 1%quantile = 30 Bq/m^3^, Q‐95%: 95% quantile = 152 Bq/m^3^, and Q‐99%: 99%‐quantile = 207 Bq/m^3^.

Figure 2 shows a comparison of all spline functions, with case probability plotted by indoor radon exposures (see Data S1, Section 4.1). We observed a J‐shaped dose–response relationship with the lowest case probability (equal to LRE) at an sIR between 38 and 64 Bq/m^3^. The mean LRE was identified at 57.6 Bq/m^3^ (95% CI: 56.1–59.2 Bq/m^3^) with a rising LC risk on both sides. The OR at an exposure level of 25 Bq/m^3^ (OR = 1.31, 95% CI: 1.01–1.59) was approximately the same as for an exposure of 100 Bq/m^3^ (OR = 1.34, 95% CI: 1.20–1.45). For an exposure level of 200 Bq/m^3^ (often defined as radon action level) we observed an OR of 1.93 (95% CI: 1.53–2.28). However, the estimated lung cancer probabilities and ORs at the very low (<25 Bq/m^3^) and high radon levels (>175 Bq/m^3^) strongly depend on the spline settings, and thus have higher uncertainty.

### 3.6 Stratified by key factors

Table 3 summarizes the results stratified by key factors including histology, smoking, sex and age (details see Data S1, sections 4.2–4.5). The risk for all histological subtypes increases with an sIR above 58 Bq/m^3^ (LRE), and most strongly for SCLC. The OR for SCLC was 2.19 (95% CI: 1.66–2.68) at 158 Bq/m^3^, compared to 2.04 (95% CI: 1.42–2.67) for large cell LC (LCLC), 1.54 (95% CI: 1.36–1.69) for AdenoLC and 1.42 (95% CI: 1.17–1.64) for SqCLC. No association was observed between sIR below 58 Bq/m^3^ and SqCL. The estimated LREs differed only slightly by histological type (SCLC, ~47 Bq/m^3^; LCLC, ~42 Bq/m^3^; and AdenoLC, ~40 Bq/m^3^).

**TABLE 3:** Selected OR estimated by subgroup.

sIR	25 Bq/m^3^		58 Bq/m^3^ (reference)	158 Bq/m^3^	
	OR	95% CI	OR = 1	OR	95% CI
By histology					
SCLC	1.14	0.46–2.24	Reference	2.19	1.66–2.68
LCLC	1.85	0.11–14.1		2.04	1.42–2.67
AdenoLC	1.22	0.81–1.64		1.54	1.36–1.69
SqCLC	0.77	0.42–1.20		1.42	1.17–1.64
By smoking					
Never smokers	2.17	1.02–3.77	Reference	2.14	1.65–2.59
Former smokers	1.04	0.67–1.43		1.77	1.51–1.99
Current smokers	1.24	0.66–1.96		1.67	1.35–1.95
By sex					
Men	1.35	0.37–3.49	Reference	1.92	1.65–2.13
Women	1.31	0.83–1.82		1.38	1.16–1.57
By age					
Age ≤ 60	1.32	0.62–2.29	Reference	1.57	1.30–1.81
Age 60–68	2.47	1.02–4.72		1.85	1.49–2.17
Age 69–74	0.74	0.35–1.28		1.48	1.14–1.79
Age 75++				2.10	1.53–2.64

*Note*: sIR: mean spatial indoor radon exposure in the surrounding area; OR: average odds ratio (reference exposure level is 58 Bq/m^3^–LRE lowest‐risk exposure; avg. by Rubin's equation with model weights derived from ΔAIC); exposure levels of 25 Bq/m^3^ and 158 Bq/m^3^ (equivalent to LRE +100 Bq/m^3^) were selected as examples; all ORs are adjusted for sex, age, smoking (type: never, former and current smoker; age start smoking; time since stop smoking; pack years); 95% CI 95% confidence interval.

The sIR‐related spatial‐dose response relationship for LC seems to vary according to the smoking status and intensity. For example, the ORs at an exposure level of 158 Bq/m^3^ are 2.14 (95% CI: 1.65–2.59), 1.77 (95% CI: 1.51–1.99) and 1.67 (95% CI: 1.35–1.95) for never, former and current smokers, respectively. Furthermore, among current smokers, the sIR conferred the stronger association among those who smoked least, with OR at an exposure level of 158 Bq/m^3^ of 1.86 (95% CI: 1.36–2.33), 1.45 (95% CI: 0.99–1.91) and 1.09 (95% CI: 0.69–1.51) for the lowest (T1), the middle (T2) and the largest (T3) pack years third. In general, current smoking appears to flatten the sIR‐related spatial‐dose response relationship, which is in line with the previously observed sub‐multiplicative but more than additive interaction between cigarette smoking and low‐dose radon exposure.^21^ A similar pattern is observed below 58 Bq/m^3^.

We observed comparable spatial‐dose response relationships between men and women within the range of 40 to 100 Bq/m^3^. However, at higher exposure level, for example, 200 Bq/m^3^, the association was stronger in men (OR = 2.00, 95%‐CI: 1.61–2.35) than in women (OR = 1.30, 95%‐CI: 0.90–1.69).

In terms of age, the spatial‐dose response relationship was J‐shaped only among those aged <69 years (*n* = 8677). Among those over 69 years of age (*n* = 5803), the sIR‐related LC risk increased steadily with increasing exposure, and was more pronounced from 200 Bq/m^3^ upward than among younger people.

Comparable non‐linear risk profiles were observed in all participating studies, most recognizable for CAPUA, EAGLE and MSH‐PMH. The profile of HSPH is flatter compared to those of the other studies, which is probably due to the choice of spouses and friends as controls. On the other hand, the US counties have an average size slightly larger than 3000 km^2^ (=48 × 48 km) and are thus 30 times larger than the 10 × 10 km grid of the European indoor radon map. This difference in size may be the cause of the observed differences in the strength of the association.

## 4 DISCUSSION

In this study, we demonstrated the utility of the newly introduced average spatial indoor radon levels as a measure of exposure to radon‐induced LC risk. It was not our aim to establish a link between radon and lung cancer for the first time, as this has already been proven by others. However, estimated bin‐wise associations based on sIR are broadly comparable to those previously reported by Krewski et al., Darby et al. and Lubin et al.,^9^, ^10^, ^11^ which are based on more precise and person‐specific exposure assessment. The semi‐parametric spline functions showed a potentially non‐linear association among those under 69 years of age but a linear spatial‐dose response for those older than 69 years. This is the largest study based on individual‐level data ever (*n* = 14,489 subjects) reported and represents one‐fifth the size of all previous studies combined.^18^ Our observation can also serve as an example of how an arbitrary choice of categorization can mask differences in risk when risk profiles are curved.

### 4.1 Use of sIR as proxy of IRC

The concentrations of naturally occurring radon in most environmental media are highly variable in time and space, even within very small areas, due to different geochemical source concentrations and radon transport factors, as well as meteorological conditions, tidal forces, tectonic and seismic events. In addition, the accumulation of radon indoors is subject to anthropogenic factors, such as the physical characteristics of a building (building materials, ventilation, drinking water sources, floor level, etc.) and occupancy and usage patterns (living habits).^28^ It is also known, that flats and detached houses represent very different radon distributions. To measure IRC in selected rooms validly and reliably in order to derive the radon exposure of a person is easy to perform, but a cost‐ and time‐intensive undertaking, because high temporal variation of IRC makes short‐term measurements unreliable for most applications.^4^, ^29^ Note that IRC averages hourly, seasonal, and yearly variations in indoor radon concentration, for example, attributable to weather changes but with a limited impact on lung cancer risk estimation^30^, ^31^ Average IRC values can even vary substantially within the same building.^32^

In the absence of measured IRC or detailed information about living conditions for each study participant, we quantified radiation exposure by radon as sIR based on publicly available data. In contrast to IRC, sIR is not only a measure of indoor radon exposure but also partially quantifies environmental radon exposure. Therefore, it is a proxy for the combination of environmental radon exposure (ER) and indoor radon exposure (IR).

The sIR was assigned based on a one‐time‐point residential address. Therefore, this is a snapshot and should not be interpreted as lifetime‐accumulated exposure as resettlement behavior is not taken into account. However, difference in this between source populations may explain some of the observed differences between the studies, as internal migration is more common in the US and Canada than in the UK, Israel, and European countries.^33^ For example, an average relocation distance of 39 km (1.9 km within cities) was given for Germany, while this is over 100 km in the much larger USA and Canada.^34^, ^35^ In summary, more frequent and far‐reaching mobility in the US and Canada may reduce the suitability of sIR as a proxy for IRC.

In addition, a year‐to‐year variability of the IRC was recognized by repeated measurements within the same house. However, the extent is inconsistent and ranges from 15% to 62% (expressed as coefficient of variance “within a house”: ratio of the standard deviation to the arithmetic mean). As a consequence, single‐point measurements of radon exposure, as the one we used, tend to bias the effect estimates of LC risk towards zero.^30^ Our OR estimates are therefore somewhat conservative, if at all.

Furthermore, although unlikely, we cannot rule out the possibility that some patients with persistent respiratory problems (which may later be diagnosed with LC) move once they discover that they live at home with high radon levels. This behavior would potentially lead to biased estimates.

Not taken into account are building characteristics, such as height, building material or energy retrofit, ventilation behavior and exposure duration. The pitfalls and reliability of IRC measurements are discussed elsewhere and are beyond the scope of this study.^4^, ^32^ We relied on IRC accuracy and validity when using the publicly available sIR values. Therefore, sIR is inferior to individual exposure assessment (IRC) for individual risk assessment. However, attempts have been undertaken by others to predict mean IRC of dwellings or estimate the probability that IRC exceeds 100 Bq/m^3^ and 300 Bq/m^3^, taking geo‐lithological and/or building characteristics into account.^32^, ^36^, ^37^, ^38^, ^39^ It is possible that in future predictions of individual IRCs will replace the community averages used here, if these are based on more finely scaled sIR values and individual living conditions can be taken into account.^40^ Until then, however, sIR appears to be suitable as a surrogate measure for IRC in large‐scale epidemiological studies.

### 4.2 Discussion on the LNT model

Our findings may contribute to the ongoing discussion on the LNT model, which originally arose from considerations by the United Nations Scientific Committee on the Effects of Radiation (UNSCEAR) and the U.S. Federal Radiation Council (FRC) in the mid‐1950s and was commonly accepted by leading radiation experts.^21^, ^41^, ^42^, ^43^ LNT has been and is still used to extrapolate spatial‐dose response curves from high to low and very low radon exposure levels to estimate health risks. LNT theoretically corresponds to a stochastic process that is associated with an increased risk of cancer through a higher number of single‐ (SSBs) and double‐strand breaks (DSBs), driven by a relatively low but dose‐dependent probability of SSB/DSBs per energy deposition event.^44^ However, such DNA damage is less likely due to natural background exposure than endogenous sources such as biologically reactive oxygen species.^45^

However, it has been suggested that a strictly linear model does not account for several biological defense processes activated by very low doses of ionizing radiation (LDR). Such processes include DNA repair, apoptosis, synthesis of heat shock proteins, free radical scavenging, bystander effects, immune stimulation, and tumor suppression.^45^, ^46^, ^47^, ^48^, ^49^

Our results show that among those younger than 69 years of age, a J‐shaped spatial‐dose response relationship is compatible with the hypothesis related to biological defense mechanisms, whereas in the older age population, the stochastic process of increasing DNA damage is more likely.

However, the observation of a non‐linear spatial‐dose response relationship for overall LC can be considered at least consistent with a similarly shaped spatial‐dose response relationship of state‐wide average indoor radon concentration (thus similar to sIR) reported for the mid‐Atlantic and northeastern US states, although observed for all‐cause mortality.^50^ Furthermore, no increased mortality from LC due to occupational radiation exposure below 200 mSv (mainly cumulative gamma radiation) was recently observed in a pooled cohort of 101,363 US nuclear power plant workers, considering data from 1944 to 2016.^51^

### 4.3 Limitations

The measurement and survey methods, and thus the quality of these sIR measures, were not standardized. This can lead to a certain degree of misclassification, especially at very low exposure levels. As often no information was provided on the accuracy of the sIR measures, we have not control for measurement errors. There was also no relationship between the time of the sIR assessment and the individual date of diagnosis. Given the long time lag between cancer initiation and clinical manifestation, this seems negligible. However, exposure misclassifications at extremely low sIR levels cannot be excluded. The weakest association observed in the HSPS study could also be because spouses and friends were recruited as controls, who usually live closer to the cases.

Finally, considering that prolonged exposure to natural radiation may induce a learned adaptive response in cells and organisms, it is unclear whether the observed spatial‐dose response relationship can be applied to regions with only low SIR exposure (e.g. Australia, the Netherlands or Florida).^21^

## 5 CONCLUSIONS

Collectively, our results provide evidence of a potentially non‐linear risk profile for radon‐induced lung cancer among populations aged <69 years, when only community‐average exposure values are available. Effect modifications due to smoking behavior and their pattern depending on the histological subtype are equivalent to those in the individual determination of radiation exposure. The assignment of spatial indoor radon levels (sIR) is an informative proxy for radon exposure and can be used in epidemiological studies; however, the accuracy of individual risk assessments needs to be further assessed.

The sIR is useful as proxy of individual radon exposure in epidemiological studies on lung cancer. The usual assumption of a linear dose–response relationship, as can be made for individual radon exposures, may not be optimal for sIR values of less than 200 Bq/m^3^.

## AUTHOR CONTRIBUTIONS

**Albert Rosenberger:** Conceptualization (equal); formal analysis (equal); methodology (equal); project administration (equal); resources (equal); writing – original draft (equal); writing – review and editing (equal). **Heike Bickeböller:** Funding acquisition (supporting); supervision (supporting). **David C. Christiani:** Resources (equal). **Geoffrey Liu:** Resources (equal). **Matthew B. Schabath:** Resources (equal); writing – review and editing (equal). **Luisa F. Duarte:** Resources (equal). **Loic Le Marchand:** Resources (equal). **Christopher Haiman:** Resources (equal). **Teresa Landi:** Resources (equal). **Dario Consonni:** Resources (equal); writing – review and editing (equal). **John K. Field:** Resources (equal). **Michael P. A. Davies:** Resources (equal). **Demetrios Albanes:** Resources (equal). **Adonina Tardón:** Resources (equal). **Guillermo Fernández‐Tardón:** Resources (equal). **Gad Rennert:** Resources (equal). **Christopher I. Amos:** Funding acquisition (equal); project administration (equal); resources (equal). **Rayjean J. Hung:** Funding acquisition (equal); project administration (equal); resources (equal); writing – review and editing (equal).

## FUNDING INFORMATION

The National Institutes of Health (7U19CA203654–02/ 397,114,564–5,111,078 Integrative Analysis of Lung Cancer Etiology and Risk) supported this work. Other individual funding sources for participating studies and members of INTEGRAL‐ILCCO are listed elsewhere [10, 30]. The funders played no role in the study design, data collection and analysis, decision to publish, or manuscript preparation. The funders had no role in study design, data collection, data analysis, or data interpretation. We gratefully acknowledge the support of the Open Access Publication Fund/Transformation Agreement of the University of Göttingen and the publication fund “NiedersachsenOPEN”.

## CONFLICT OF INTEREST STATEMENT

The authors have no relevant financial or non‐financial interests to disclose.

## ETHICS STATEMENT

The participating studies were approved by the respective internal review board or ethics committee. Informed consent was obtained from all the participants included in the studies. All consortium research was approved by the Dartmouth Committee for the Protection of Human Subjects on 7/30/2014 (id STUDY00023602).

## Supporting information


Data S1:

## Data Availability

Indoor radon values for Canada are publicly available at https://open.canada.ca/data/en/dataset/744d8a3b‐b9e0‐41b8‐be5f‐5f869a36a221. State Radon Map Books for the United States are available at https://nepis.epa.gov/. The data that support the findings of this study are available from ILCCO/INTEGRAL from the authors upon reasonable request and with permission from the ILCCO/INTEGRAL data access committee.
