# Supplementary material for: On the informative value of community‐based indoor radon values in relation to lung cancer
Source: Cancer Med. 2024 Aug 28;13(16):e70126. doi: 10.1002/cam4.70126 (PMC11350838; doi:10.1002/cam4.70126)
Supplement: Supplementary file 1 — Data S1: [file CAM4-13-e70126-s001.docx]

**Supporting Information**

[1 ILCCO participating Studies included in this analysis 2](#_Toc137816464)

[Sources of spatial indoor radon exposure (sIR) 3](#_Toc137816465)

[2 Methods (Details) 4](#_Toc137816466)

[2.1 Source or indoor radon values and assignment to study participants 4](#_Toc137816467)

[2.2 Spatial indoor radon exposure in the surrounding area: main response variable sIR 4](#_Toc137816468)

[2.3 Spatial indoor radon exposure (sIR): Distribution 4](#_Toc137816469)

[2.4 Spatial indoor radon exposure (sIR): Extreme values 4](#_Toc137816470)

[*3* *Definition of parametric models* 5](#_Toc137816471)

[3.1 Spline models 5](#_Toc137816472)

[4 Results of parametric risk models 7](#_Toc137816473)

[5 Results of spline models (ΔAIC-weighted) 8](#_Toc137816474)

[5.1.1 Overall lung cancer 8](#_Toc137816475)

[5.1.2 Subgroups by histology 8](#_Toc137816476)

[5.1.3 Subgroups by smoking 9](#_Toc137816477)

[5.1.4 Subgroups by sex and age 11](#_Toc137816478)

[5.1.5 Subgroups by study site 12](#_Toc137816479)

[6 References 13](#_Toc137816480)

[Supplementary Table 1 Data contribution ILCCO studies 2](#_Toc137816422)

[Supplementary Table 2 Source or sIR and assignment to study participants 3](#_Toc137816423)

[Supplementary Table 3 Missing summary measures for sIR in the surrounding area 4](#_Toc137816424)

[Supplementary Table 4 Fit of heavy tail distribution 4](#_Toc137816425)

[Supplementary Table 5 excess odds ratio (EOR) by parametric risk models 7](#_Toc137816426)

[Supplementary Table 6 Odds ratio estimates for overall LC 8](#_Toc137816427)

[Supplementary Table 7 Odds ratio estimates by histology 8](#_Toc137816428)

[Supplementary Table 8 Odds ratio estimates by smoking 9](#_Toc137816429)

[Supplementary Table 9 Odds ratio estimates by smoking group and amount 10](#_Toc137816430)

[Supplementary Table 10 Odds ratio estimates by sex and age 11](#_Toc137816431)

[Supplementary Table 11 Odds ratio estimates by study site 12](#_Toc137816432)

[Supplementary Figure 1 Odds ratio estimates by histology 9](#_Toc137816346)

[Supplementary Figure 2 Odds ratio estimates by smoking group 10](#_Toc137816347)

[Supplementary Figure 3 Odds ratio estimates by smoking group and amount 10](#_Toc137816348)

[Supplementary Figure 4 Odds ratio estimates by sex and age 11](#_Toc137816349)

[Supplementary Figure 5 Odds ratio estimates by participating study 12](#_Toc137816350)

# ILCCO participating Studies included in this analysis

Supplementary Table 1 Data contribution ILCCO studies

| Study Name (lang) | Study acronym | PI | Country | Year | Control eligibility/ recruitment | Matching factors |
| --- | --- | --- | --- | --- | --- | --- |
| CAncer de PUlmon en  Asturias | CAPUA | A. Tardon | Spain | 2002‐2012 | resident, no previous cancer | sex |
| Environment And Genetics in Lung cancer Etiology | EAGLE | M.T. Landi | Italy | 2002‐2005 | health registry | age, sex |
| Harvard Lung Cancer Study | HSPH | D.C. Christiani | US | 1992‐2004 | friends/spouse | none |
| Israel Lung Cancer Study | NICCC-LCA | G. Rennert | Israel | 2005-present | Population registry | age, sex, clinic |
| Mount-Sinai Hospital-Princess Margaret Study | MSH-PMH | R. Hung / G. Liu | Canada | 2008‐2012 | patients registered at the family medicine clinic | age, sex |
| International Early Lung  Cancer Action Program | IELCAP-Toronto | G. Liu | Canada | 2004-2011 | >10 pack years, >50 yrs | age, sex, smoking |

Only ILCCO studies with sufficient sIR variability with more than 10 cases or controls with sIR over 50 Bq/m³ were included in the analysis. The participating studies of ILCCO are individually described in the supplement of McKay et al. (1).

# Sources of spatial indoor radon exposure (sIR)

Supplementary Table 2 Source or sIR and assignment to study participants

|  | provider of sIR data | data source | ref. | data grid | survey years | linked LC-study | recruitment year | address link to sIR data |
| --- | --- | --- | --- | --- | --- | --- | --- | --- |
| Canada | Radiation Health Assessment Division (RHAD), Health Canada - Radiation Protection Bureau | electronic record ^$^ | (2) | counties and 3-diget post codes | 2009-2011 | MSH-PMH | 2008‐2012 | by 3-diget postal codes |
| Israel | Soreq Nuclear Research Center | electronic report (Hebrew version) | (3) | counties (**שם האזור)** | 1998-2012 | NICCC-LCA | 2005- | by Natural Region |
| Italy | Centro Nazionale per la Protezione dalle Radiazioni e Fisica Computazionale | electronic record | (4,5) | counties (comune) | 1989-1998 2004-2007 | EAGLE | 2002‐2005 | by county names |
| Spain | --- | European Indoor radon map | (4,5) | 10x10 km grid | 1989-2010 | CAPUA | 2002‐2012 | by geo-coordinates |
| United Kingdom | Radon Group, Public Health England (PHE) | electronic record | (5,6) | 10x10 km grid | 1980-2005 | LLP | 1999‐2016 | via postal codes and geo-coordinates ^$$^ |
| United States of America | US Environmental Protection Agency (EPA) | EPA’s state radon map book ^§^ | (7) | counties / cities | 1986-1992 | HSPH MEC TLC | 1992‐2004 1993‐1996 2012‐ | by county names ^&&^ equal sIR values ^&^ by county names ^&&^ |

sIR spatial indoor radon; ref. reference, geolocation: A tool for converting geo-coordinates coordinates into grid cells of the European indoor radon map was provided to us by P. Bossew from the Federal Office for Radiation Protection, Berlin; ^&^ Since there is almost no radon exposure in Hawaii, all participants were assigned the same value. ^&&^ taking the US states into account. ^$$^ postal codes were transformed into geo-coordinates using <https://www.doogal.co.uk/UKPostcodes.php>; ^$^ publically available at <https://open.canada.ca/data/en/dataset/744d8a3b-b9e0-41b8-be5f-5f869a36a221>; ^§^ publically available at <https://nepis.epa.gov/>; Study name abbreviations: see **Fehler! Verweisquelle konnte nicht gefunden werden.**.

# Methods (Details)

## Source or indoor radon values and assignment to study participants

Source of sIR-value: We obtained regional indoor radon exposure (IR) data from representative, cross-regional or nationwide survey in dwellings, including the USA, Canada, Israel and the United Kingdom. IR data for Spain had to be extracted from the European Indoor radon map ([https://remap.jrc.ec.europa.eu/Atlas.aspx#](https://remap.jrc.ec.europa.eu/Atlas.aspx))(4). Details are provided in Supplement2.

Linkage to residential location: Information on place of residence upon diagnosis up to partial postal codes, natural region code, or county were available from the participating ILCCO studies, in accordance to the ethics and privacy policy set by the study institutions. The residential information was blindly linked to sIR values obtained from the survey, which provides the mean spatial indoor radon exposure in the surrounding area for each study participant.

For studies in the US, Canada, Italy and Israel, the residential location was linked to sIR by postal/ZIP or Natural-Region codes, or by county or community/township names. Geo-coordinates (longitudes and latitudes) for these locations were retrieved (<https://www.geonames.org/>). For other European studies, geo-coordinates (longitudes and latitudes) of the residential location were first determined using GPS visualizer (<http://www.gpsvisualizer.com/geocoder/>). These were then transformed into grid cells of the European indoor radon map using a tool personally provided to us by the Federal Office for Radiation Protection, Berlin. All links have been in turn plotted on geographic maps to verify their unambiguous location in the catchment areas of the ILCCO studies and in inhabited areas. When necessary, the mapping was repeated to obtain optimal data quality. If the place of residential information was incomplete, ambiguous, or inaccurate, residency was assigned to a next higher administrative unit (e.g., to a region instead of a municipality, or 2-digit instead 3-digit postal code).

## Spatial indoor radon exposure in the surrounding area: main response variable sIR

| Supplementary Table 3 Missing summary measures for sIR in the surrounding area | | | | | | | | | | | |
| --- | --- | --- | --- | --- | --- | --- | --- | --- | --- | --- | --- |
| Study site |  | mean | | median | | geom.mean | | mean of ln | | US radon zone | |
|  | N | NMiss | % | NMiss | % | NMiss | % | NMiss | % | NMiss | % |
| CAPUA | 718 | 2 | 0% | 2 | 0% | 2 | 0% | 2 | 0% | 718 | 100% |
| EAGLE | 1.618 | 30 | 2% | 30 | 2% | 1618 | 100% | 1618 | 100% | 1618 | 100% |
| HSPH | 2.295 | 11 | 0% | 253 | 11% | 213 | 9% | 2295 | 100% | 124 | 5% |
| NICCC-LCA | 583 | 2 | 0% | 583 | 100% | 583 | 100% | 583 | 100% | 583 | 100% |
| MSH-PMH | 1.394 | 17 | 1% | 1394 | 100% | 1394 | 100% | 1394 | 100% | 1394 | 100% |
| *total* | *6608* | *62* | *1%* | *2262* | *34%* | *3810* | *58%* | *5892* | *89%* | *4437* | *67%* |
| NMiss number of missing values; geom.mean geometric mean, US United States of America | | | | | | | | | | | |

There have been up to five summary measures of *spatial indoor radon exposure in the surrounding area* (IR) extracted from available surveys including mean, median, geometric mean, mean of logarithmic values and US radon zone, which are strongly correlated with each other (all ρ>0.7). Only the *mean of IR of the surrounding region* could be extracted with a minimum of missing values (in 325 of 8.256 links, 4%). Hence, the mean spatial indoor radon values were considered as *sIR*, the main response variable for the analysis (see S2-Table 8).

## Spatial indoor radon exposure (sIR): Distribution

sIR values of representative dwelling samples are known to follow a skewed and heavy tailed distribution. The fitting of inter alia lognormal or gamma distributions to spatial indoor radon values have been discussed^[[1]](#footnote-2)^ (8–11). However, sIR values can only be assumed as roughly log-normal distributed within windows up to a few 10 km radius (and under some other conditions) and after outliers or extremes have been removed (9). We found the log-normal distribution to fit best to the observed sIR values.

Supplementary Table 4 Fit of heavy tail distribution

| Distribution | converted | KS | selected |
| --- | --- | --- | --- |
| **Gamma** | yes | 17.27470 | no |
| **Burr** | yes | 19.12046 | no |
| **Weibull** | yes | 17.19577 | no |
| **Logn** | yes | **15.10954** | **yes** |
| **Gpd** | yes | 28.74406 | no |

KS Kolmogorov-Smirnov Statistic

## Spatial indoor radon exposure (sIR): Extreme values

Extreme values of $sIR$ may represent unrealistic proxies of individual exposure, due to the spatial sampling process within dwellings, and may introduce bias during parameter estimation. Thus, we replaced values exceeding a threshold of 259 Bq/m³ by the respective quantiles of a log-normal-distribution for more robust modelling (8,9).

## Definition of parametric models

The linear non-threshold model (**LNT**) is defined as:

$\frac{\pi}{1-\pi}=(1+\beta_{r}r)e^{\beta_{0}+\boldsymbol{\beta X}}$ (Equation 1)

From this definition follows, that the odds ratio is $OR=(1+\beta_{r}r$), where $\beta_{r}$ is referred as excess odds ratio (EOR). $\beta_{r}$ can be interpreted as the percentage change of OR by one unit of the exposure r. $e^{\beta_{0}+\boldsymbol{\beta X}}$ can be considered as baseline odd.

Per definition, a continuous increase (decrease) in the risk is modelled the further the exposure r is different from 0. An exposure of r=0 (even if not exists) is considered as the “lowest (largest) risk exposure level” (LRE).

We also fitted a modified version of LNT (referred as **LNT+**), incorporating a shift parameter $\beta_{t}$.

The linear shifted non-threshold model (LNT+) is defined as:

$\frac{\pi}{1-\pi}=(1+\beta_{r}(r-\beta_{t}))e^{\beta_{0}+\boldsymbol{\beta X}}$ (Equation 2)

The reference exposure level corresponding to the baseline odd (respectively an OR=1) is then$r-\beta_{t}$.

The linear threshold model (**LT**) is defined as:

$\frac{\pi}{1-\pi}=\begin{matrix} (1+\beta_{r}(r-\beta_{t}))e^{\beta_{0}+\boldsymbol{\beta X}} & r>\beta_{t} \\ e^{\beta_{0}+\boldsymbol{\beta X}} & r\leq\beta_{t} \end{matrix}$ (Equation 3)

In contrast to the LNT, the lowest (largest) risk is assigned to every individual with exposure r$\leq\beta_{t}$; a continuous increase (decrease) in the risk is modelled the further the exposure r exceeds $\beta_{t}$.

The linear mirror point model (further referred as **LT+**) is defined as:

$\frac{\pi}{1-\pi}=\begin{matrix} (1+\beta_{r}(r-\beta_{t}))e^{\beta_{0}+\boldsymbol{\beta X}} & r>\beta_{t} \\ e^{\beta_{0}+\boldsymbol{\beta X}} & r=\beta_{t} \\ (1{+\beta}_{r}(\beta_{t}-r))e^{\beta_{0}+\boldsymbol{\beta X}} & r<\beta_{t} \end{matrix}$ (Equation 4)

Here, the LRE is considered to be at $\beta_{t}$, with continuous increase (decrease) in the risk the further the exposure r differs from $\beta_{t}$.

## Spline models

To overcome the limitation of the linearity assumption of the parametric models, we fit logistic regression model with spline functions (piecewise polynomial functions) for sIR. The degree (dg) of the piecewise polynomial functions is set in advancedSAS PROC LOGISTIC provides the fit of B-splines, where additional (so-called boundary) knots are placed automatically outside the range of pre-specified knots. We also fitted natural cubic splines, where the function is linear beyond the extreme knots. It is known that the choice of the pre-defined internal knots and the “spline basis” change the model fit (12).

Spline settings

In M=44 spline models, we positioned internal knots either a) by typical radon thresholds (e.g. 50, 100 or 200 Bq/m³), b) as “equally spaced knots” in terms of exposure quantiles (e.g. then 25%-, 50%- and 75%-quartiles) or c) according to a recommendation by Harrell et al. (12). ‘We further fit B-splines (Bsp) and natural cubic splines (NCS).

Modelling strategy

We next applied the following strategy, which results in model averaging.

1. *Fitting splines*: We applied the $M=44$ different spline definitions (*sp* for model $m$) within the framework of logistic regression and one basic model without a spline for sIR: $\ln\left( \frac{\pi}{1-\pi} \right)=\alpha+{sp}_{m}\left( sIR \right)+\boldsymbol{\beta X}$
   with $\pi$ the case-probability within the sample, ${sp}_{m}$ the spline-function for sIR, and $\boldsymbol{\beta X}$ to adjust for other covariates.
   The model (spline) fit was assess by a range of Bayesian-type generalized information criteria $BGIC(c,r)$ ($i$ indexing a set $(c,r)$); including the specification of Akaike information criterion AIC=BGIC(c=2, r=0) and Bayesian information criterion BIC=BGIC(c=1, r=1)).
2. *Deriving weights*: In order to average over all splines ${sp}_{m}\left( sIR \right)$, instead of lasting on a single model, we searched for weights $w_{m,i}$ from those $BGIC(c,r)$, which provides the lowest concentration (quantified by Hirschman-Herfindahl-Index ${HHI}_{i}$. Details see below.
   The ΔAIC (difference in AIC of models with and without a spline for sIR) was found to be almost optimal for model weighting for the overall LC as well as for the considered subgroups. Hence, ΔAIC-based results are reported.
3. *Lowest risk exposure level (LRE)*: The sIR at the minimum case-probability of an estimated spline function ${sp}_{m}\left( sIR \right)$ (if some exist between 15 and 120 Bq/m³) is considered as the *lowest risk exposure level* (*LRE_m_*). These were averaged over all spline models. This weighted mean is presented as LRE along with the 95% prediction intervals and the range. This was applied to the total sample (for overall LC) and in subgroups.
4. *Model specific odds ratios*: ${OR}_{m,sIR}$ estimates were derived for each “spline model” $m$ at pre-defined exposure levels (e.g. $sIR$=100 Bq/m³), relative to the reference exposer of LRE at 58 Bq/m³.
5. *Average odds ratios*: We finally averaged the corresponding ${OR}_{m,sIR}$ estimates to $\bar{OR}_{sIR}$applying a weighted version Rubin’s equation (13,14). Hence, in constructing 95% confidence intervals for ${OR}_{sIR}$ we took into account estimating and modelling uncertainty.

We fitted the spline functions adjusted for sex, age and smoking (type: never, former and current smoker; age at smoking initiation; time since stop smoking; pack years). We repeated the analysis in subgroups by sex, age (defined by quartiles), smoking type (never smokers; former and current smokers split into thirds by pack years), participating study, and by histological subtype (SCLC, SqCLC, LCLC and AdenoLC).

Deriving Odds ratios from spline function

According to the logistic regression framework, we calculated the odds ratio at exposure level sIR relative to a reference value at 58 Bq/m³ (median in the total sample), as ${OR}_{m,sIR}=\frac{{odd}_{m,sIR}}{{odd}_{m.64 Bq/m^{3}}}=e^{{sp}_{m}\left( sIR \right)-{sp}_{m}(64)}$, with ${sp}_{m}\left( sIR \right)$ the spline-function *m*.

Definition of $\mathrm{BGIC}$, with $\mathrm{AIC}$ and $\mathrm{BIC}$ as special case

The *B*ayesian-type *G*eneralized *I*nformation *C*riteria is defined as $BGIC(c,r)=-D\left( \theta\right)+c\cdot d\cdot{(logN)}^{r}$ (15), where $D\left( \theta\right)$is the model deviance ($D\left( \theta\right)=2\log\left( p\left( \boldsymbol{y} | \theta\right) \right)$), d is the number of free parameters in the model and N is the sample size. Setting c=2 and r=0 results in $BGIC=AIC=-D\left( \theta\right)+2d$. Setting c=1 and r=1 results in $BGIC=BIC=-D\left( \theta\right)+d(logN)$. We calculated $BGIC(c,r)$ for r= 0 to 2 by 0.5 and c=0 to 3 by 0.5, differently weighting the number of free parameters, respectively the sample size.

Deriving model weights $w_{m,i}$

We estimate model weights (as recommended (14)) by $\hat{w}_{m,i}=\frac{e^{-\Delta{{BGIC}_{m,i}}/2}}{\sum_{k=1}^{M} e^{-\Delta{{BGIC}_{k,i}}/2}}$, where M is the number of “spline models”, and - to correct for the model fit attributable to covariates - ${\Delta BGIC}_{m,i}={BGIC}_{basis,i}-{BGIC}_{m,i}$ with ${BGIC}_{basis}(c,r)$ the corresponding information criteria of the model without spline for sIR. Alternative weights were calculated without correction by ${BGIC}_{basis,i}$.

Concentration of model weights $w_{m,i}$: Herfindahl-Hirschman Index

For each $BGIC(c,r)$ we calculated the Herfindahl-Hirschman Index ${HHI}_{i}=\frac{\sum_{m=1}^{M} w_{i,m}^{2}-1/M}{1-1/M}$, (16) as concentration measure for distributions of discrete random variables with M possible realisations. HHI ranges between 0 (all $w_{i,m}=\frac{1}{M}$, minimal concentration since all spline are given the same weight) and 1 (one $w_{i,m}=1$ all other 0, maximal concentration in the case only one of all splines is selected).
For the weighting of splines, preference should be given to those BGIC(c,r) criteria whose weights are distributed as evenly as possible over all splines (lower HHI). In other words, the final OR estimates should not rely on a single or a few fitted spline functions.

Weighted Rubin’ equation

We applied a weighted adaption (17) of Rubin method (13) on pooling (averaging) estimates over multiple samples for on the same date on the odds ratio estimate

Consider $\theta_{m}$ to represent the Odds ratio estimate ${OR}_{m,sIR}$ derived from spline *m* at an exposure level of *sIR*.

The model-averaged value $\bar{\theta}$ can be estimated as $\hat{\bar{\theta}}=\sum_{m=1}^{M} \hat{w}_{m}\hat{\theta}_{m}$,
under the condition of $\sum_{m=1}^{M} \hat{w}_{m}=M$.

The sampling variance of the estimator of $\bar{\theta}$ can be derived from the between sample variance (representing model uncertainty) and then within sample variance (representing estimation uncertainty).

The between sample variance B can be estimated as $B=\frac{1}{M}\sum_{m=1}^{M} \hat{w}_{m}(\hat{\theta}_{m}-{\hat{\bar{\theta}}}_{m})^{2}$.

Then average within sample variance V can be calculated as $\bar{V}=\sum_{m=1}^{M} \hat{w}_{m}s.e(\hat{\theta}_{m})^{2}$.

The variance estimate associated with $\hat{\bar{\theta}}$ is the total variance $T=\bar{V+\left( 1+\frac{1}{M} \right)B}$.

The statistic ($\theta-\hat{\bar{\theta}})T^{-\frac{1}{2}}$ T follows approximately $\sim t_{v_{m}}$, with $v_{m}=(M-1)\left[ 1+\frac{\bar{V}}{\left( 1+\frac{1}{M} \right)B} \right]^{2}$ degrees of freedom.

Prediction interval for LRE

Since the lowest turning point of each U-shaped spline considered as LRE, we present the mean LRE along with the 95% prediction interval (95% PI). This interval contains with 95% probability the LRE of an arbitrary spline function fitted to data of a sample of the same size, drawn from the same initial population and analyzed in the same way.

(In contrast, a 95% confidence interval is that range that covers the true LRE at 95%, in a sample of the same size, drawn from the same initial population and analyzed in the same way).

Given $n=n_{u}+n_{n.u}$ spline-function, of which $n_{u}$ are u-shaped, $n_{n.u}$ are not, ${LRE}_{i}$ tuning-points are derived $(i=1\ldots n_{u}).$ Of this the mean LRE $\bar{LRE}$ and the standard deviation $s_{LRE}$ can be calculated, the standard error ${s.e.}_{LRE}={s_{LRE}}/{\sqrt{n_{u}-1}}$ can be derived. Usually, the 95% PI, assuming normal distributed LRE values, is defined as:

$\bar{LRE} \pm t\cdot({s.e.}_{LRE}+s_{LRE})\equiv\bar{LRE} \pm t\cdot s_{LRE}\cdot(\frac{1+\sqrt{n_{u}-1}}{\sqrt{n_{u}-1}})$ (Equation 5)

(with ${s.e.}_{LRE}$ representing the uncertainty in the estimation of the true LRE by the $LRE$; and $s_{LRE}$ representing the variability between the spline-specific LRE-values, an $t$ being the 97,5%-quantile of a t-distribution with ${df=n}_{u}-1$ degrees of freedom)

To account for the fact that only $n_{u}$ instead of $n$ LRE-values could be derived, we replace $s_{LRE}$by $s_{LRE}^{*}=\sqrt{n-1}\cdot{s.e.}_{LRE}=\sqrt{\frac{n-1}{n_{u}-1}}\cdot s_{LRE}$. ( $s_{LRE}^{*}$ representing the variability between the spline-specific LRE-values by accounting that only $n_{u}$ values could be observed.)

Hence calculated the adapted 95% PI as

$\bar{LRE} \pm t\cdot({s.e.}_{LRE}+s_{LRE}^{*})\equiv\bar{LRE} \pm t\cdot s_{LRE}\cdot(\frac{1+\sqrt{n-1}}{\sqrt{n_{u}-1}})$ (Equation 6)

For robustness, we calculates unweighted 95% PIs. The minimal lower interval borders were set to zero. Please note, due to accounting for the number of splines without a minimum, the conservative 95% PI can be wider than the observed range on LREs among u-shaped splines.

# Results of parametric risk models

Supplementary Table 5 excess odds ratio (EOR) by parametric risk models

| fitted model | sIR [Bq/m³] | LRE/REL |  | adj. EOR | 95% CI |
| --- | --- | --- | --- | --- | --- |
| LNT | per 100 Bq/m³ | 0 Bq/m³ | by definition | 0.58 | +0.37 - +0.80 |
| Darby et al. | per 100 Bq/m³ | 0 Bq/m³ | by definition | 0.08 | +0.03 - +0.16 |
| Krewski et al. | per 100 Bq/m³ | 0 Bq/m³ | by definition | 0.10 | ─0.01 - +0.26 |
| Li et al. | per 100 Bq/m³ | 0 Bq/m³ | by definition | 0.11 | +0.05 - +0.17 |
| LNT+ | per 100 Bq/m³ | 25 Bq/m³ | REL estimated | 0.51 | ─1.34 - +2.36 |
| LT | per 100 Bq/m³ | 66 Bq/m³ | LRE estimated | 0.62 | +0.43 - +0.80 |
| LT+ | per 100 Bq/m³ | 47 Bq/m³ | LRE estimated | 0.57 | +0.39 - +0.75 |

adj. EOR excess odds ratio per 100 Bq/m³ adjusted for study site, sex, age, smoking (type: never, former and current smoker; age at smoking initiation; time since stop smoking; pack years); 95% CI 95% confidence interval; LNT linear non-threshold model; LNT+ linear shifted non-threshold model; LT linear threshold model; LT+ linear mirror point model (model equations are given in chapter 2.5); REL reference exposure level (at which OR=1), LRE lowest risk exposure

# Results of spline models (ΔAIC-weighted)

## Overall lung cancer

Supplementary Table 6 Odds ratio estimates for overall LC

|  | OR | 95% CI | p-value | betw./total var. | # models |
| --- | --- | --- | --- | --- | --- |
| **10 Bq/m³** | 1.66 | 0.90-2.59 | 0.0977 | 0% | 44 |
| **20 Bq/m³** | 1.41 | 1.03-1.78 | 0.0285 | 1% | 44 |
| **25 Bq/m³** | **1.31** | **1.01-1.59** | **0.0385** | 1% | 44 |
| **30 Bq/m³** | 1.22 | 0.99-1.42 | 0.0582 | 1% | 44 |
| **40 Bq/m³** | 1.09 | 0.97-1.20 | 0.1333 | 2% | 44 |
| **50 Bq/m³** | 1.05 | 0.98-1.10 | 0.1307 | 3% | 44 |
| **55 Bq/m³** | 1.01 | 0.99-1.02 | 0.2406 | 4% | 44 |
| **58 Bq/m³** | **1.00** | **reference** |  |  | 44 |
| **75 Bq/m³** | 1.10 | 1.04-1.14 | 0.0010 | 6% | 44 |
| **80 Bq/m³** | 1.14 | 1.07-1.20 | 0.0001 | 5% | 44 |
| **100 Bq/m³** | **1.34** | **1.20-1.45** | **<.0001** | 5% | 44 |
| **150 Bq/m³** | 1.65 | 1.48-1.78 | <.0001 | 1% | 44 |
| **164 Bq/m³** | 1.70 | 1.52-1.85 | <.0001 | 1% | 44 |
| **200 Bq/m³** | **1.93** | **1.53-2.28** | <.0001 | 1% | 44 |
| **250 Bq/m³** | 2.34 | 1.24-3.72 | 0.0083 | 1% | 44 |

sIR: mean spatial indoor radon exposure in the surrounding area; OR: averaged odds ratios (reference class: 58 Bq/m³; avg. by Rubin’s equation with model weights derived from the Akaike information criterion ΔAIC); all ORs are adjusted for sex, age, smoking (type: never, former and current smoker; age start smoking; time since stop smoking; pack years); 95% CI 95% confidence interval; betw./total var.: Percentage of between point estimate variance on the total variance; # model: number of models that were averaged

## Subgroups by histology

Supplementary Table 7 Odds ratio estimates by histology

| sIR | SqCLC | | SCLC | | LCLC | | AdenoLC | |
| --- | --- | --- | --- | --- | --- | --- | --- | --- |
|  | OR | 95% CI | OR | 95% CI | OR | 95% CI | OR | 95% CI |
| **10 Bq/m³** | 1.03 | 0.34-2.32 |  |  |  |  |  |  |
| **20 Bq/m³** | 0.77 | 0.42-1.20 | 1.14 | 0.44-2.27 |  |  | 1.62 | 1.09-2.16 |
| **25 Bq/m³** | 0.86 | 0.53-1.24 | 1.14 | 0.46-2.24 | 1.85 | 0.11-14.1 | 1.22 | 0.81-1.64 |
| **30 Bq/m³** | 0.97 | 0.67-1.27 | 1.24 | 0.65-1.97 | 1.23 | 0.44-2.61 | 1.02 | 0.74-1.29 |
| **40 Bq/m³** | 0.95 | 0.77-1.10 | 0.86 | 0.59-1.13 | 0.83 | 0.51-1.18 | 1.04 | 0.90-1.17 |
| **50 Bq/m³** | 1.00 | 0.90-1.08 | 0.56 | 0.40-0.71 | 0.86 | 0.68-1.02 | 1.15 | 1.05-1.23 |
| **55 Bq/m³** | 1.00 | 0.96-1.03 | 0.80 | 0.71-0.88 | 0.97 | 0.91-1.02 | 1.05 | 1.01-1.07 |
| **58 Bq/m³ (reference)** | **1.00** | **reference** | **1.00** | **reference** | **1.00** | **reference** | **1.00** | **reference** |
| **75 Bq/m³** | 1.12 | 1.01-1.20 | 1.44 | 1.22-1.63 | 1.04 | 0.86-1.20 | 1.00 | 0.94-1.05 |
| **80 Bq/m³** | 1.17 | 1.04 | 1.27 | 1.49 | 1.25 | 1.69 | 1.06 | 0.84-1.25 |
| **100 Bq/m³** | 1.35 | 1.13-1.54 | 1.64 | 1.33-1.91 | 1.15 | 0.85-1.42 | 1.19 | 1.07-1.28 |
| **150 Bq/m³** | 1.41 | 1.19-1.60 | 2.07 | 1.61-2.50 | 1.80 | 1.32-2.25 | 1.50 | 1.33-1.63 |
| **158 Bq/m³ (ref.+100)** | **1.42** | **1.17-1.64** | **2.19** | **1.66-2.68** | **2.04** | **1.42-2.67** | **1.54** | **1.36-1.69** |
| **200 Bq/m³** | 1.81 | 0.79-3.32 | 2.21 | 1.23-3.40 |  |  | 1.90 | 1.49-2.27 |
| **250 Bq/m³** |  |  |  |  |  |  | 2.40 | 1.13-4.14 |

sIR: mean spatial indoor radon exposure in the surrounding area; OR: averaged odds ratios (reference class: 58 Bq/m³ -LRE lowest risk exposure; avg. by Rubin’s equation with model weights derived from ΔAIC); all ORs are adjusted for sex, age, smoking (type: never, former and current smoker; age start smoking; time since stop smoking; pack years); 95% CI 95% confidence interval; LCLC: large cell lung cancer; SCLC: small cell lung cancer; SqCLC: squamous cell lung cancer, AdenoLC: lung adenocarcinoma

Supplementary Figure 1 Odds ratio estimates by histology


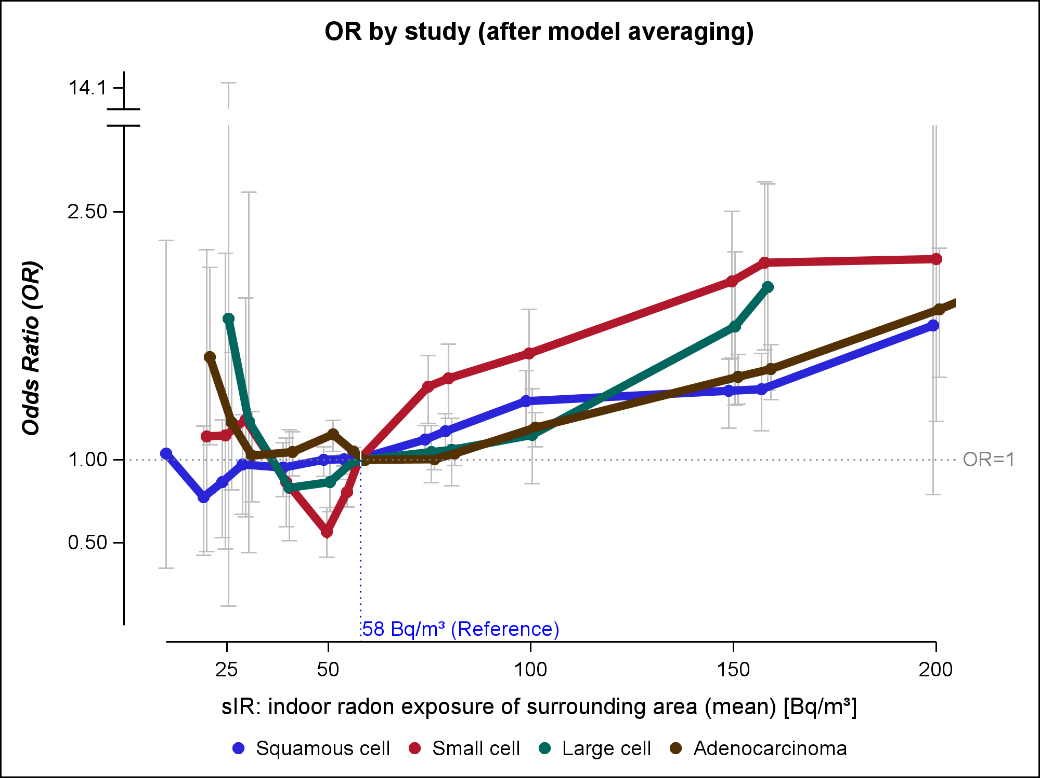


## Subgroups by smoking

Supplementary Table 8 Odds ratio estimates by smoking

| sIR | never smokers | | former smokers | | current smokers | |
| --- | --- | --- | --- | --- | --- | --- |
|  | OR | 95% CI | OR | 95% CI | OR | 95% CI |
| **20 Bq/m³** | 2.67 | 0.75-6.76 | 1.08 | 0.58-1.72 | 1.40 | 0.69-2.37 |
| **25 Bq/m³** | 2.17 | 1.02-3.77 | 1.04 | 0.67-1.43 | 1.24 | 0.66-1.96 |
| **30 Bq/m³** | 1.66 | 0.96-2.47 | 1.00 | 0.72-1.27 | 1.15 | 0.72-1.63 |
| **40 Bq/m³** | 1.32 | 1.00-1.62 | 0.94 | 0.79-1.06 | 1.07 | 0.85-1.26 |
| **50 Bq/m³** | 1.12 | 0.96-1.25 | 0.95 | 0.88-1.00 | 1.12 | 0.98-1.24 |
| **55 Bq/m³** | 1.02 | 0.97-1.05 | 0.98 | 0.95-0.99 | 1.04 | 0.99-1.07 |
| **58 Bq/m³ (reference)** | **1.00** | **reference** | **1.00** | **reference** | **1.00** | **reference** |
| **75 Bq/m³** | 1.14 | 1.01-1.25 | 1.20 | 1.10-1.28 | 1.00 | 0.90-1.08 |
| **80 Bq/m³** | 1.20 | 1.04-1.35 | 1.27 | 1.14-1.37 | 1.02 | 0.91-1.12 |
| **100 Bq/m³** | 1.48 | 1.18-1.74 | 1.51 | 1.29-1.70 | 1.17 | 1.00-1.31 |
| **150 Bq/m³** | 2.04 | 1.60-2.44 | 1.74 | 1.50-1.93 | 1.61 | 1.32-1.85 |
| **158 Bq/m³ (ref.+100)** | 2.14 | 1.65-2.59 | 1.77 | 1.51-1.99 | 1.67 | 1.35-1.95 |
| **200 Bq/m³** | 2.74 | 1.45-4.36 | 1.68 | 1.05-2.38 | 2.10 | 1.19-3.18 |

sIR: mean spatial indoor radon exposure in the surrounding area; OR: averaged odds ratios (reference class: 58 Bq/m³ -LRE lowest risk exposure; avg. by Rubin’s equation with model weights derived from ΔAIC); all ORs are adjusted for sex, age, smoking (type: never, former and current smoker; age start smoking; time since stop smoking; pack years); 95% CI 95% confidence interval

Supplementary Figure 2 Odds ratio estimates by smoking group


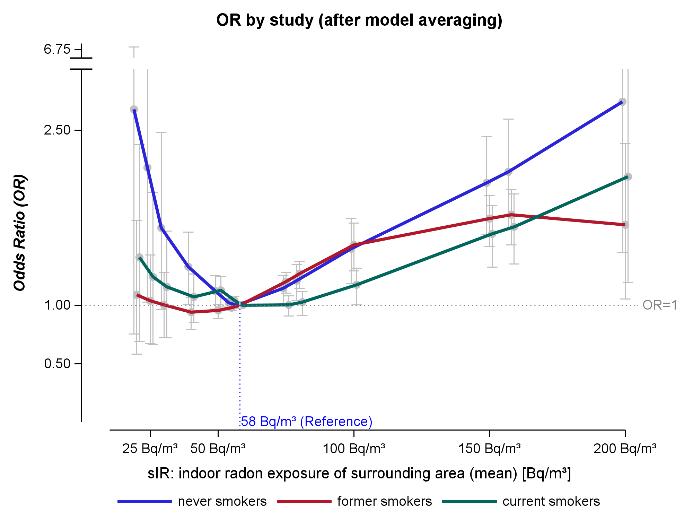


Supplementary Table 9 Odds ratio estimates by smoking group and amount

| sIR | current smokers  (T1) | | current smokers (T2) | | current smokers  (T3) | | former smokers (T1) | | former smokers (T2) | | former smokers (T3) | |
| --- | --- | --- | --- | --- | --- | --- | --- | --- | --- | --- | --- | --- |
|  | OR | 95% CI | OR | 95% CI | OR | 95% CI | OR | 95% CI | OR | 95% CI | OR | 95% CI |
| **20 Bq/m³** |  |  |  |  |  |  |  |  |  | 0.05-1.27 |  |  |
| **25 Bq/m³** |  |  |  |  |  |  | 1.34 | 0.63-2.34 | 0.55 | 0.19-1.20 |  |  |
| **30 Bq/m³** |  |  |  |  |  |  | 0.98 | 0.58-1.44 | 0.79 | 0.35-1.44 |  |  |
| **40 Bq/m³** | 1.17 | 0.79-1.55 | 1.37 | 0.72-2.18 | 0.93 | 0.50-1.45 | 0.99 | 0.78-1.17 | 0.94 | 0.62-1.26 | 0.76 | 0.37-1.28 |
| **50 Bq/m³** | 1.04 | 0.87-1.18 | 1.19 | 0.89-1.46 | 1.24 | 0.89-1.58 | 0.99 | 0.90-1.06 | 1.01 | 0.82-1.18 | 0.82 | 0.64-0.99 |
| **55 Bq/m³** | 1.01 | 0.95-1.05 | 1.06 | 0.96-1.14 | 1.06 | 0.95-1.15 | 0.99 | 0.96-1.01 | 1.00 | 0.93-1.06 | 0.94 | 0.87-1.00 |
| **58 Bq/m³ (reference)** | **1.00** | **reference** | **1.00** | **reference** | **1.00** | **reference** | **1.00** | **reference** | **1.00** | **reference** | **1.00** | **reference** |
| **75 Bq/m³** | 1.06 | 0.91-1.18 | 0.98 | 0.82-1.12 | 0.91 | 0.71-1.09 | 1.17 | 1.05-1.26 | 1.14 | 0.94-1.30 | 1.09 | 0.88-1.28 |
| **80 Bq/m³** | 1.09 | 0.92-1.24 | 1.00 | 0.81-1.17 | 0.90 | 0.68-1.12 | 1.24 | 1.09-1.36 | 1.19 | 0.95-1.41 | 1.10 | 0.85-1.33 |
| **100 Bq/m³** | 1.27 | 1.00-1.51 | 1.11 | 0.83-1.37 | 0.93 | 0.64-1.21 | 1.53 | 1.24-1.78 | 1.36 | 1.00-1.71 | 1.07 | 0.76-1.38 |
| **150 Bq/m³** | 1.78 | 1.32-2.20 | 1.40 | 0.99-1.82 | 1.08 | 0.72-1.47 | 1.65 | 1.37-1.90 | 1.66 | 1.17-2.14 | 1.17 | 0.79-1.57 |
| **158 Bq/m³ (ref.+100)** | 1.86 | 1.36-2.33 | 1.45 | 0.99-1.91 | 1.09 | 0.69-1.51 | 1.65 | 1.34-1.92 | 1.80 | 1.19-2.45 | 1.27 | 0.81-1.76 |
| **200 Bq/m³** | 2.24 | 1.32-3.30 | 1.67 | 0.80-2.85 | 0.87 | 0.36-1.67 | 1.56 | 0.82-2.50 |  |  |  |  |

sIR: mean spatial indoor radon exposure in the surrounding area; Q1 lowest, Q2 middle and Q3 largest pack years thirds; OR: averaged odds ratios (reference class: 58 Bq/m³ -LRE lowest risk exposure; avg. by Rubin’s equation with model weights derived from ΔAIC); all ORs are adjusted for sex, age, smoking (type: never, former and current smoker; age start smoking; time since stop smoking; pack years); 95% CI 95% confidence interval

Supplementary Figure 3 Odds ratio estimates by smoking group and amount


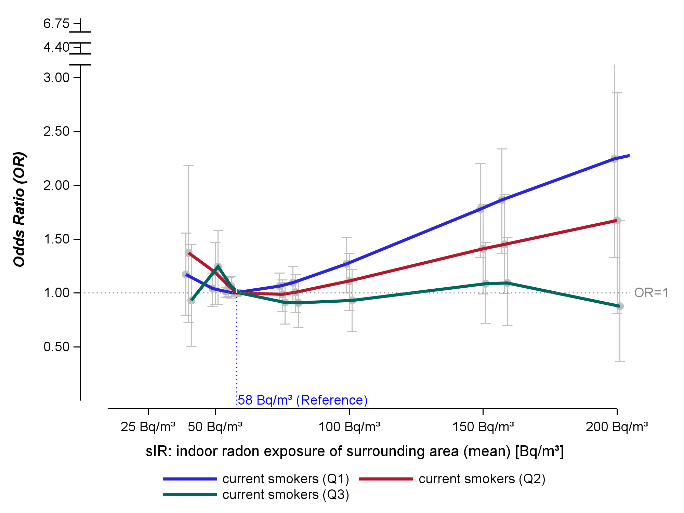

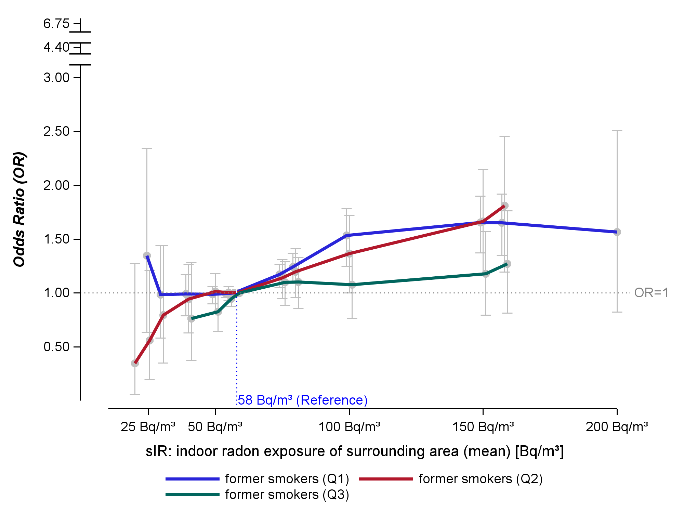


## Subgroups by sex and age

Supplementary Table 10 Odds ratio estimates by sex and age

| sIR | age ≤60 | | age 60-68 | | age 69-74 | | age 75++ | | men | | women | |
| --- | --- | --- | --- | --- | --- | --- | --- | --- | --- | --- | --- | --- |
|  | OR | 95% CI | OR | 95% CI | OR | 95% CI | OR | 95% CI | OR | 95% CI | OR | 95% CI |
| **20 Bq/m³** | 3.68 | 0.80-11.2 | 3.10 | 0.59-10.4 | 0.72 | 0.22-1.71 |  |  |  |  | 1.25 | 0.78-1.78 |
| **25 Bq/m³** | 1.32 | 0.62-2.29 | 2.47 | 1.02-4.72 | 0.74 | 0.35-1.28 |  |  | 1.35 | 0.37-3.49 | 1.31 | 0.83-1.82 |
| **30 Bq/m³** | 0.86 | 0.47-1.35 | 1.87 | 1.10-2.76 | 0.80 | 0.45-1.22 | 0.78 | 0.02-10.5 | 1.06 | 0.65-1.52 | 1.27 | 0.86-1.68 |
| **40 Bq/m³** | 1.48 | 1.13-1.80 | 1.21 | 0.95-1.45 | 0.93 | 0.72-1.13 | 0.61 | 0.43-0.78 | 1.05 | 0.88-1.19 | 1.09 | 0.90-1.26 |
| **50 Bq/m³** | 1.22 | 1.05-1.37 | 1.06 | 0.94-1.16 | 0.98 | 0.87-1.06 | 0.87 | 0.74-0.98 | 1.08 | 0.97-1.16 | 1.00 | 0.92-1.06 |
| **55 Bq/m³** | 1.04 | 0.99-1.08 | 1.01 | 0.97-1.04 | 0.99 | 0.95-1.02 | 0.97 | 0.92-1.00 | 1.01 | 0.98-1.04 | 0.99 | 0.96-1.01 |
| **58 Bq/m³ (reference)** | **1.00** | **reference** | **1.00** | **reference** | **1.00** | **reference** | **1.00** | **reference** | **1.00** | **reference** | **1.00** | **reference** |
| **75 Bq/m³** | 1.08 | 0.97-1.17 | 1.17 | 1.04-1.27 | 1.05 | 0.94-1.15 | 1.07 | 0.93-1.18 | 1.10 | 1.03-1.16 | 1.10 | 1.00-1.18 |
| **80 Bq/m³** | 1.13 | 1.00-1.24 | 1.24 | 1.08-1.37 | 1.07 | 0.93-1.19 | 1.09 | 0.92-1.23 | 1.15 | 1.06-1.23 | 1.14 | 1.02-1.25 |
| **100 Bq/m³** | 1.31 | 1.10-1.49 | 1.53 | 1.24-1.78 | 1.15 | 0.94-1.34 | 1.22 | 0.96-1.44 | 1.38 | 1.22-1.51 | 1.31 | 1.10-1.49 |
| **150 Bq/m³** | 1.54 | 1.28-1.76 | 1.83 | 1.49-2.13 | 1.40 | 1.10-1.67 | 1.90 | 1.43-2.34 | 1.87 | 1.62-2.07 | 1.40 | 1.19-1.58 |
| **158 Bq/m³ (ref.+100)** | 1.57 | 1.30-1.81 | 1.85 | 1.49-2.17 | 1.48 | 1.14-1.79 | 2.10 | 1.53-2.64 | 1.92 | 1.65-2.13 | 1.38 | 1.16-1.57 |
| **200 Bq/m³** | 1.61 | 1.10-2.13 | 1.86 | 1.23-2.51 | 2.19 | 1.07-3.69 | 3.63 | 1.51-6.90 | 2.00 | 1.61-2.35 | 1.30 | 0.90-1.69 |
| **250 Bq/m³** |  |  | 1.98 | 0.69-4.27 | 4.67 | 0.39-28.5 | 5.22 | 1.28-14.6 |  | 1.40-2.62 |  |  |

sIR: mean spatial indoor radon exposure in the surrounding area; OR: averaged odds ratios (reference class: 58 Bq/m³ -LRE lowest risk exposure; avg. by Rubin’s equation with model weights derived from ΔAIC); all ORs are adjusted for sex, age, smoking (type: never, former and current smoker; age start smoking; time since stop smoking; pack years); 95% CI 95% confidence interval

Supplementary Figure 4 Odds ratio estimates by sex and age


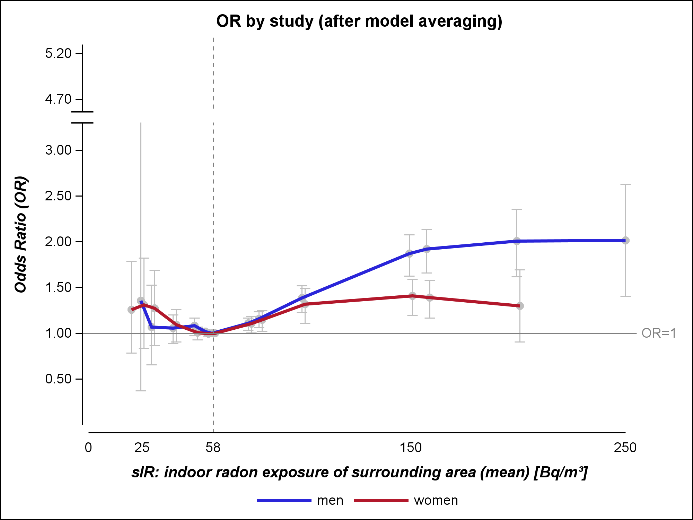

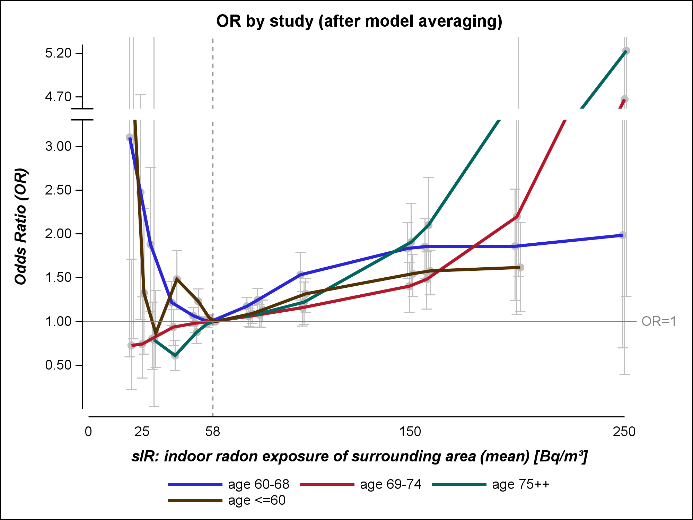


## Subgroups by study site

Supplementary Table 11 Odds ratio estimates by study site

| sIR | CAPUA | | EAGLE | | HSPH | | NICCC-LCA | | MSH-PMH | |
| --- | --- | --- | --- | --- | --- | --- | --- | --- | --- | --- |
|  | OR | 95%CI | OR | 95% CI | OR | 95% CI | OR | 95% CI | OR | 95% CI |
| **20 Bq/m³** |  |  |  |  | 1.04 | 0.55-1.65 |  |  |  |  |
| **25 Bq/m³** |  |  |  |  | 0.96 | 0.62-1.33 |  |  |  |  |
| **30 Bq/m³** | 1.73 | 0.11-12.8 |  |  | 0.92 | 0.62-1.22 |  |  |  |  |
| **40 Bq/m³** | 2.28 | 0.07-28.3 | 1.23 | 0.90-1.56 | 0.91 | 0.68-1.14 | 1.02 | 0.56-1.57 | 1.02 | 0.57-1.58 |
| **50 Bq/m³** | 1.28 | 0.21-4.73 | 1.17 | 0.92-1.40 | 0.96 | 0.85-1.06 | 1.07 | 0.75-1.39 | 0.65 | 0.42-0.89 |
| **55 Bq/m³** | 1.06 | 0.49-1.84 | 1.03 | 0.93-1.11 | 0.98 | 0.94-1.02 | 1.02 | 0.88-1.13 | 0.47 | 0.29-0.66 |
| **58 Bq/m³ (reference)** | **1.00** | **reference** | **1.00** | **reference** | **1.00** | **reference** | **1.00** | **reference** | **1.00** | **reference** |
| **75 Bq/m³** | 1.25 | 0.08-8.82 | 1.22 | 0.92-1.49 | 1.01 | 0.85-1.14 | 1.39 | 0.55-2.73 | 6.53 | 2.65-12.6 |
| **80 Bq/m³** | 1.31 | 0.09-9.10 | 1.32 | 1.01-1.61 | 1.01 | 0.83-1.17 | 1.88 | 0.26-7.86 | 3.67 | 1.49-7.08 |
| **100 Bq/m³** | 1.38 | 0.10-9.20 | 1.98 | 1.40-2.56 | 1.05 | 0.83-1.24 |  |  | 5.82 | 1.09-19.8 |
| **150 Bq/m³** |  |  |  |  | 0.96 | 0.79-1.10 |  |  |  |  |
| **158 Bq/m³ (ref.+100)** |  |  |  |  | 0.96 | 0.79-1.12 |  |  |  |  |
| **200 Bq/m³** |  |  |  |  | 1.15 | 0.58-1.90 |  |  |  |  |

sIR: mean spatial indoor radon exposure in the surrounding area; OR: averaged odds ratios (reference class: 58 Bq/m³ -LRE lowest risk exposure; avg. by Rubin’s equation with model weights derived from ΔAIC); all ORs are adjusted for sex, age, smoking (type: never, former and current smoker; age start smoking; time since stop smoking; pack years); 95% CI 95% confidence interval;

Supplementary Figure 5 Odds ratio estimates by participating study


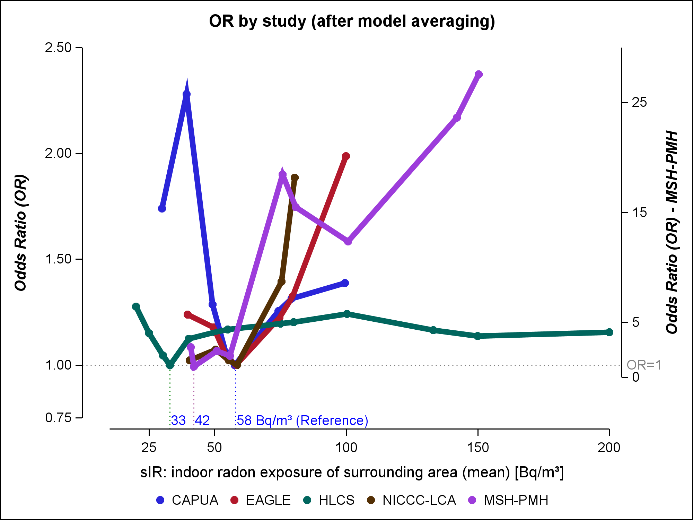


sIR: mean spatial indoor radon exposure in the surrounding area; all ORs are adjusted for sex, age, smoking (type: never, former and current smoker; age start smoking; time since stop smoking; pack years) and averaged by Rubin’s equation with model weights derived from ΔAIC; ORs for study specific reference class: 33,42 or 58 Bq/m³ - OR for MSH-PMM scaled differently.

# References

1. McKay JD, Hung RJ, Han Y, Zong X, Carreras-Torres R, Christiani DC, u. a. Large-scale association analysis identifies new lung cancer susceptibility loci and heterogeneity in genetic susceptibility across histological subtypes. Nat Genet. Juli 2017;49(7):1126–32.

2. Cross-Canada Survey of Radon Concentrations in Homes - Final Report [Internet]. Ottawa, Canada: Health Canada; 2012 [zitiert 10. August 2018]. Verfügbar unter: www.healthcanada.gc.ca

3. G. Haquin, T. Riemer, Y. Shamai, M. Margaliot, M. Shirav-Schwartz, R. Kenett. Radon Survey of Israel [Internet]. 2012. Verfügbar unter: https://www.academia.edu/52597665/Radon_Survey_of_Israel

4. Tollefsen T, Cinelli G, Bossew P, Gruber V, De Cort M. From the European indoor radon map towards an atlas of natural radiation. Radiation Protection Dosimetry. 2014;162(1–2):129–34.

5. European Commission, Joint Research Centre, Čeliković I, Vukanac I, Gruber V, Nikolić J, u. a. Literature review of indoor radon surveys in Europe. Publications Office; 2019.

6. Grainger P, Shalla SH, Preece AW, Goodfellow SA. Home radon levels and seasonal correction factors for the Isle of Man. Phys Med Biol. August 2000;45(8):2247–52.

7. EPA’s Map of Radon Zones [Internet]. United States Environmental Protection Agency; Office of Radiation and Indoor Air (ORIA); 1993. Report No.: 402-R-93–021 to 402-R-93–070. Verfügbar unter: https://nepis.epa.gov/

8. Murphy P, Organo C. A comparative study of lognormal, gamma and beta modelling in radon mapping with recommendations regarding bias, sample sizes and the treatment of outliers. J Radiol Prot. August 2008;28(3):293–302.

9. Bossew P. Radon: exploring the log-normal mystery. J Environ Radioact. Oktober 2010;101(10):826–34.

10. Tuia D, Kanevski M. Indoor radon distribution in Switzerland: lognormality and Extreme Value Theory. Journal of Environmental Radioactivity. 1. April 2008;99(4):649–57.

11. Daraktchieva Z, Miles JCH, McColl N. Radon, the lognormal distribution and deviation from it. J Radiol Prot. März 2014;34(1):183–90.

12. Harrell FE. Regression modeling strategies with applications to linear models, logistic regression, and survival analysis. New York Berlin Heidelberg: Springer Verlag; 2001. (Springer series in Statistics).

13. Rubin DB. Multiple Imputation for Nonresponse in Surveys [Internet]. Wiley & Sons Ltd; 2004. 258 S. (Wiley Classics Library). Verfügbar unter: https://www.wiley.com/en-us/Multiple+Imputation+for+Nonresponse+in+Surveys-p-9780471655749

14. Wang H, Zhang X, Zou G. Frequentist model averaging estimation: a review. J Syst Sci Complex. Dezember 2009;22(4):732–48.

15. Lu ZH. On an Expression of Generalized Information Criterion [Internet]. Workshop on current trends and challenges in Model Selection and Related Areas; 2008 Juli 24; Vienna, Austria. Verfügbar unter: https://www.univie.ac.at/workshop_modelselection/slides/lu/lu_slides.pdf

16. Bleymüller J, Gehlert G, Gülicher H. Statistik für Wirtschaftswissenschaftler. 15., überarb. Aufl. München: Vahlen; 2008. 246 S. (WiSt-Studienkurs).

17. Burnham KP, Anderson DR. Model Selection and Multimodel Inference: A Practical Information-Theoretic Approach [Internet]. 2. Aufl. New York: Springer-Verlag; 2002. Verfügbar unter: https://www.springer.com/gp/book/9780387953649

1. [↑](#footnote-ref-2)
